# Can exoscope improve brain AVMs surgery?

**DOI:** 10.3171/2023.10.FOCVID23114

**Published:** 2024-01-01

**Authors:** Serena Tola, Federico Capelli, Alessandro Della Puppa

**Affiliations:** Department of Neuroscience, Neurosurgery Clinic, Psychology, Pharmacology and Child Health (NEUROFARBA), Careggi University Hospital and University of Florence, Italy

**Keywords:** arteriovenous malformation, AVM, exoscope, indocyanine green video angiography, ICG-VA, microsurgery

## Abstract

The advantages of the surgical view provided by the exoscope have been described before, although reports of its application to brain arteriovenous malformation (AVM) surgery are lacking. The ampler field of view and magnification up to ×24 allow for enhanced visualization during microsurgical procedures. Furthermore, the live visualization provided by indocyanine green video angiography (ICG-VA) helps emphasize the hemodynamics of AVMs, even allowing the detection of possible residual vein arterialization as an indirect expression of nidal remnants. With this illustrative video showing the resection of a hemorrhagic right frontoinsular Spetzler-Martin grade III AVM, the authors describe the technical implications of exoscope brain AVM surgery using the Olympus ORBEYE 4K-3D, with a final focus on ICG-VA as an asset.

The video can be found here: https://stream.cadmore.media/r10.3171/2023.10.FOCVID23114

## Transcript

### 0:25 Case Presentation.

A 46-year-old man with established diagnosis of brain AVM presented to the emergency department with symptoms of sudden hemiparesis and a rapid neurological deterioration to GCS 4 requiring orotracheal intubation. The CT scan revealed a right frontoparietal hemorrhage with ventricular hemorrhage, as well as signs of the former endovascular treatments. He underwent emergency external ventricular drainage with ICP microsensor.

### 0:55 Digital Subtraction Angiography and Treatment Strategy.

DSA showed a Spetzler-Martin grade III right frontoinsular AVM. Feeders came from right MCA with a deep insular feeder as well. A single superficial venous drainage toward the sylvian middle cerebral vein was present. Treatment strategy consisted of a preliminary embolization of the deepest feeders, followed by surgical excision and hematoma evacuation.

DSA and CT-angio performed after the preoperative embolization show nidus reduction and slowdown of the venous drainage.

### 1:35 Surgical Approach.

Surgery was performed under exoscope view. Exoscope view was preferred to gather a more immersive 3D intraoperative view, thus allowing the surgical team to share exactly the same view of the surgeon and bringing to a prompter interaction. Moreover, the possibility to watch ICG-VA live was found to be more informative and fastening surgery progression, particularly when repeated ICG-VA may be required.^1^

Patient was in supine position with the head turned toward the left of 45°. The head was fixed in the Mayfield’s clamp. A frontotemporal incision and craniotomy were performed. Neuronavigation and intraoperative neurophysiological monitoring for somatosensory evoked potential (SSEP) and motor evoked potential (MEP) were also employed.

### 2:22 Dural Incision.

Initial dural incision was tailored with neuronavigation away from the AVM’s nidus, to obtain cerebral release by partially evacuating the cerebral hemorrhage.

### 2:33 First ICG-VA.

ICG-VA after dural opening showed a dark area on the frontal lobe where signs of the former embolization were detectable, thus further aiding the AVM’s nidus localization. ICG-VA also clearly showed the arterialized draining vein with anterograde flow toward the superficial middle cerebral vein.

### 2:52 AVM Dissection and Isolation.

Nidus dissection started on the most anterior part of the AVM toward a small corticectomy and proceeded circumferentially to completely isolate the most superficial portion of the nidus.

The posterior aspect of the nidus disclosed the intracerebral hematoma.

Advancing in the dissection of the deeper portion of the AVM, several feeders were cauterized starting from the smaller and then shifting to greater ones. Former embolized vessels were isolated and cut. The draining vein side of the nidus was also isolated. The hematoma evacuation was almost completed, thus facilitating the final nidus delimitation.

Exoscope allowed to repeatedly change the angle of view during nidus dissection without relevant changes in the surgeon position, thus resulting in an enhanced ergonomics for both the surgeon and assistant. Furthermore, the compact camera frame preserved from hampering both the field of view and surgical movements.

### 4:34 Final ICG-VA and AVM Removal.

After completing dissection, the nidus was finally pulled out and an ICG-VA was performed before closing the venous drainage. ICG-VA showed some residual arterialized flux in the vein. Thus, a tiny portion of the nidus, strictly adherent to the drainage vein, was dissected and excluded. The vein was closed and the AVM was removed.

### 5:27 ICG-VA Venous Arterialization.

Since some residual vein arterialization is an indirect expression of nidal remnants,^2–4^ a last ICG-VA was performed after AVM removal. Even this ICG-VA showed persistent vein arterialization. Thus, we further dissected the more distal part of the drainage, unveiling a tiny artery resulting in an AV fistula.

### 5:57 Final ICG-VA.

Exoscope allowed to perform an ICG-VA even at high magnification without relevant losing of image definition.^1^ ICG-VA confirmed the presence of the AV fistula, which was then further dissected along with a small nidus remnant adherent to the hidden wall of the vein, which was then removed. No more vein arterialization was detectable at the following ICG-VA.

Exoscope provided for a wide range of motion and field of view. Learning curve isn’t steep and surgical movements’ adaptivity takes advantage mostly from operating on superficial lesions.^1^ As a consequence, surgical movements (even clip positioning) were not significantly affected during surgery.

### 7:19 Postoperative DSA.

Postoperative DSA showed complete removal of the AVM with no residual shunts. The patient partially recovered, and he was discharged to rehabilitation with an mRS of 4.

## Disclosures

The authors report no conflict of interest concerning the materials or methods used in this study or the findings specified in this publication.

## Author Contributions

Primary surgeon: Della Puppa. Assistant surgeon: Tola. Editing and drafting the video and abstract: Tola, Capelli. Critically revising the work: all authors. Reviewed submitted version of the work: Tola, Capelli. Approved the final version of the work on behalf of all authors: Tola. Supervision: Tola, Della Puppa.

## Supplemental Information

### Patient Informed Consent

Patient informed consent was not obtained in this study. The patient was comatose and therefore could not provide consent to treatment.
